# Spontaneous Perineal Trauma during Non-Operative Childbirth—Retrospective Analysis of Perineal Laceration Risk Factors

**DOI:** 10.3390/ijerph19137653

**Published:** 2022-06-23

**Authors:** Grażyna Bączek, Ewa Rzońca, Dorota Sys, Sylwia Rychlewicz, Anna Durka, Patryk Rzońca, Agnieszka Bień

**Affiliations:** 1 Department of Obstetrics and Gynecology Didactics, Faculty of Health Sciences, Medical University of Warsaw, 00-575 Warsaw, Poland; erzonca@wum.edu.pl (E.R.); anna.durka@wum.edu.pl (A.D.); 2 Department of Reproductive Health, Centre of Postgraduate Medical Education, 01-004 Warsaw, Poland; dorota.sys@cmkp.edu.pl; 3 St. Sophia’s Specialist Hospital, Żelazna Medical Center, 01-004 Warsaw, Poland; s.rychlewicz@szpitalzelazna.pl; 4 Department of Human Anatomy, Faculty of Health Sciences, Medical University of Warsaw, 02-004 Warsaw, Poland; patryk.rzonca@wum.edu.pl; 5 Chair of Obstetrics Development, Faculty of Health Sciences, Medical University of Lublin, 20-081 Lublin, Poland; agnieszka.bien@umlub.pl

**Keywords:** childbirth, risk factors, perineum, laceration

## Abstract

Childbirth-related perineal trauma (CRPT) is defined as damage to the skin, muscles of the perineum, as well as to the anal sphincter complex and anal epithelium. The aim of the study was to analyze the risk factors for spontaneous injuries to the soft tissues of the birth canal during non-operative delivery. This was a single-center retrospective case-control study. The study included the analysis of two groups, the study group featured 7238 patients with spontaneous perineal laceration (any degree of perineal laceration) and the control group featured patients without perineal laceration with 7879 cases. The analysis of single-factor logistic regression showed that the factors related to perineal laceration during childbirth are the age of the patients giving birth (*p* = 0.000), the BMI before delivery (*p* = 0.000), the number of pregnancies (*p* = 0.000) and deliveries (*p* = 0.000), diagnosed gestational diabetes (*p* = 0.046), home birth (*p* = 0.000), vaginal birth after cesarean (VBAC) (*p* = 0.001), the use of oxytocin in the second stage of childbirth (*p* = 0.041), the duration of the second stage of childbirth (*p* = 0.000), body weight (*p* = 0.000), and the circumference of the newborn head (*p* = 0.000). Independent factors that increase the risk of perineal laceration during childbirth are an older age of the woman giving birth, a history of cesarean section, a higher birth weight of the newborn, and factors that reduce the risk of spontaneous perineal trauma are a higher number of deliveries and home birth.

## 1. Introduction

Labor is one of the most important and unique events in the life of a woman and her relatives [1]; however, it is associated with the risk of negative maternal outcomes, such as trauma to the perineum experienced by women during vaginal delivery [1,2]. The perineum in women is the area of soft tissue that extends from the anus to the quadrilateral area of the vulva—the posterior border of the vulvar vestibule. During pregnancy, there is an increase in blood flow in this area, and during vaginal delivery—overstretching can cause trauma [3]. It should be emphasized that 85% of women experience a perineal injury, and at least one-third of them experience spontaneous perineal laceration [3,4].

Childbirth-related perineal trauma (CRPT) is defined as damage to the skin, muscles of the perineum, as well as to the anal sphincter complex and anal epithelium. This injury occurs because of spontaneous laceration or incision of the perineum during vaginal delivery [4]. There are four degrees of a perineal laceration that can be distinguished. The first degree involves damage to the skin of the perineum and vaginal mucosa. The second degree involves damage to the perineal muscles, but without the anal sphincter. The third degree involves damage to the perineum and the sphincter complex. The fourth degree involves the anal sphincter complex and anal epithelium [2,5,6,7]. The factors that increase the risk of perineal trauma include several maternal, neonatal, and intrapartum determinants [2,6,8,9].

Trauma of the perineum during childbirth may be associated with numerous health consequences of varying duration and, at the same time, on women’s quality of life. These consequences include, inter alia, perineal pain, wound dehiscence, infections, dyspareunia and sexual dysfunctions, and urinary and fecal incontinence [1,4,8,10]. Moreover, perineal trauma, perineal pain, and prolonged labor are important factors associated with a higher level of postpartum fatigue [11]. It should be noted that trauma to the perineum during childbirth and its numerous negative consequences for women’s health pose a challenge for midwives and obstetricians during delivery. Knowledge and experience, as well as preparation for the second stage of labor, are extremely important to minimize the risk of perineal trauma [6,12]. That is why the work of Tsakiridis et al. (2018) reviewed the differences between the recommendations of the Royal College of Obstetricians and Gynaecologists, the American College of Obstetricians and Gynecologists, and the Society of Obstetricians and Gynecologists of Canada regarding the prevention and treatment of obstetric anal sphincter injury (OASIS). It was emphasized that routine incision of the perineum in the prevention of OASIS is not recommended, whereas warm compresses of the perineum and massage of the perineum in the second stage of labor appear to be protective [13].

It should be emphasized that in the literature on the subject, there are numerous reports of spontaneous perineal trauma, in particular, severe perineal trauma. Perineal lacerations of the third and fourth degree are the most severe forms of spontaneous perineal trauma, and at the same time, are associated with numerous negative and long-term consequences for women’s health [5,9,13,14,15,16,17,18,19,20,21,22,23,24,25].

However, the issue of perineal lacerations (considering all degrees) is not analyzed so widely, which was the basis for our research. In addition, deepening and analyzing factors affecting spontaneous perineal injuries during childbirth allows for the verification of current guidelines for childbirth management; therefore, the aim of the study was to analyze the risk factors for spontaneous injuries to the soft tissues of the birth canal during non-operative delivery.

## 2. Materials and Methods

### 2.1. Study Groups

This was a single-center retrospective case-control study. Strobe guidelines for case-control studies were used to ensure proper reporting of results [26]. The electronic patient records of Saint Sophia’s Hospital in Warsaw, Poland, a tertiary hospital with the largest number of deliveries per year both in the city of Warsaw and in the Mazowieckie Voivodeship (the largest and most populous of the 16 Polish provinces), were used to create an anonymous retrospective database of all deliveries from 1 January 2015 to 31 December 2020.

Deliveries at Saint Sophia’s Hospital take place in two areas, the hospital delivery room or the birth house, which is an intermediate form between inpatient delivery and home birth. This dataset was generated using electronic medical records collected by medical personnel; therefore, there is no recall bias. Additionally, the dataset was cross-checked for inconsistencies and any detected were verified. Data on the woman giving birth, the course of childbirth, and the condition of the newborn are recorded in a computer database by midwives during and immediately after Childbirth The first perineal injury assessment is performed by the midwife. The first- and second-degree injuries and the incision are surgically treated by the midwife. In the case of major injuries, they ask a doctor for consultation and the doctor sutures the perineum. 

An analysis of the documentation covering 40,007 deliveries at the analyzed time was carried out. The study included the analysis of two groups, the study group of patients with spontaneous perineal laceration, any degree of perineal laceration: I—6910 cases, II—308 cases, III and IV—20 cases (due to the very small number of cases of third- and fourth-degree lacerations, they were merged together), and the control group of patients without perineal laceration. Patients who had an episiotomy at labor were excluded from the analysis.

The inclusion criterion for the study group was the occurrence of one of the perineal traumas during labor, first-, second-, third-, or fourth-degree perineal laceration. On the other hand, the inclusion criterion for the control group was the absence of any perineal trauma during labor (spontaneous or perineal incision).

**Figure 1:**
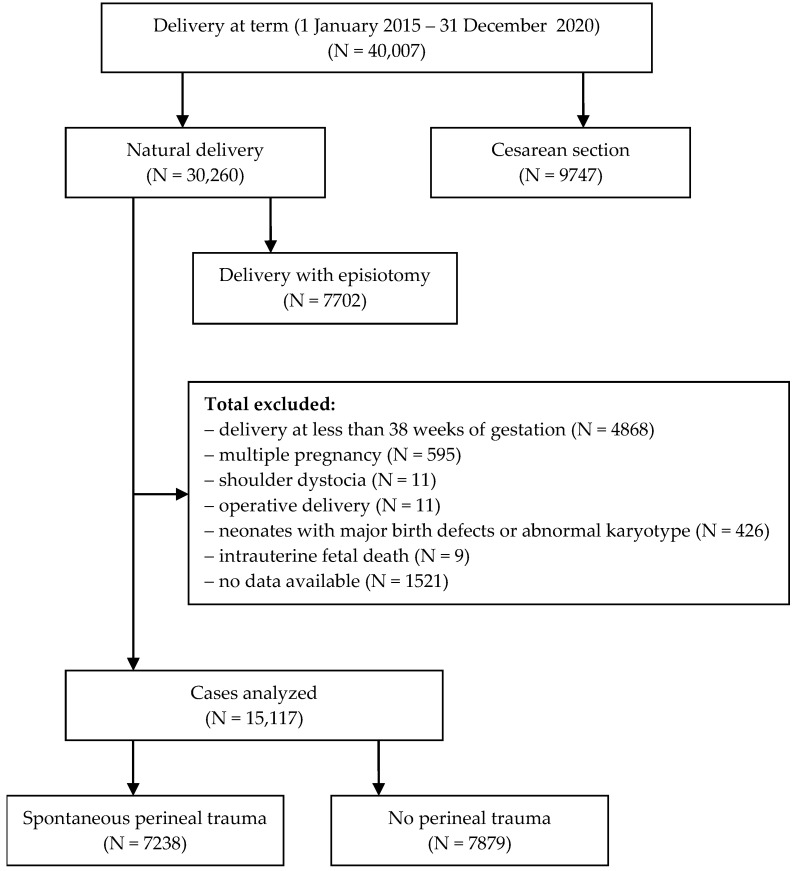
Flow diagram of exclusions and final analytic sample.

Deliveries before 38 weeks of gestation, multiple pregnancies, the occurrence of shoulder dystocia, operative deliveries, neonates with major birth defects or abnormal karyotype, and intrauterine fetal death were also excluded from the analysis. The analysis also did not consider data in which there were deficiencies in the medical records. Based on the adopted criteria, 15,117 cases were qualified for the analysis: study group—7238 cases; control group—7897 cases (Figure 1).

In the process of analyzing electronic documentation, the following information was obtained: demographic data, obstetric history, course and complications of pregnancy, diseases coexisting with pregnancy, the course of childbirth, conduct of labor, and delivery data.

The study has received approval from the Bioethics Committee of the Medical University of Warsaw (No. AKBE/204/2021). This was a retrospective anonymized data analysis; therefore, no individual patient consent was needed.

### 2.2. Statistical Analysis

The data obtained in the documentation analysis process were subjected to statistical analysis, which was performed using the R language in the RStudio environment. Qualitative data are presented as numbers (n) and case percentages (%). Quantitative data are presented as mean (M) and standard deviation (SD). The normality of the distribution of quantitative variables was checked using the Kolmogorov–Smirnov test and the Lilliefors test. The Pearson’s chi-squared test was used to assess the dependence within the qualitative variables. Quantitative variables were compared using the Student’s T-test with the assessment of homogeneity of variance with the Brown Forsythe test. A logistic regression model was developed to assess risk factors for a perineal laceration. The backward stepwise method was used in the construction. Model data are presented as odds ratios (ORs) together with the 95% confidence interval (95% CI). The usefulness of the model was assessed using the ROC method with the determination of the cutoff point by the tangent method. The level of statistical significance was set at *p* < 0.05.

## 3. Results

The conducted statistical analysis showed that the examined women with spontaneous perineal laceration were older (M = 31.9) and had a higher BMI (27.4 vs. 26.9) than in the control group. They had higher education (89.6%) and were in a stable relationship (85.1%) more often, compared to the control group. Subjects who experienced perineal laceration were more likely to be pregnant for the first time (29.9%), primiparous (35.1%), and diagnosed with gestational diabetes (8.9%). The above-mentioned relationships were statistically significant (*p* < 0.05). There was no relationship between the occurrence of perineal rupture and the place of residence, pregnancy hypertension, or pregnancy cholestasis (*p* > 0.05). Detailed data are shown in Table 1.

The spontaneous perineal laceration in the respondents occurred more often when they gave birth in a hospital (81.2%), with vaginal birth after cesarean (VBAC) (4.9%), they received oxytocin in the second stage of childbirth (0.8%). The duration of the second stage of labor was longer (23.4 vs. 21.6 min) compared to the control group. Neonates of mothers who had perineal laceration more often received 7 or more points on the Apgar scale in the first minute after delivery (99.3%). In addition, these neonates had a higher birth weight (3529.1 vs. 3464.4 grams) and head circumference (34.8 vs. 34.6 cm) compared to neonates of mothers who did not have the perineal laceration. The indicated correlations were statistically significant (*p* < 0.05). There was no relationship between the occurrence of perineal laceration and the use of oxytocin in the first stage of labor, the administration of oxytocin in the first and second stage of labor, the performance of epidural anesthesia, the duration of the first stage of labor and the Apgar score of the neonate in 5th and 10th minute (*p* > 0.05)—Table 2.

In patients of higher age, the first-degree perineal laceration occurred more often (M = 31.9); in younger patients (M = 29.9), spontaneous third- and fourth-degree perineal trauma. In women who were pregnant for the first time and during the first childbirth, the second-degree perineal laceration (pregnancy—59.7%; childbirth—65.9%) and third- and fourth-degree (pregnancy—70.0%; childbirth—75.0%) perineal trauma occurred more often. In the second pregnancy and during the second childbirth (pregnancy—43.0%; childbirth—49.5%) and in the third and subsequent one (pregnancy—28.6%; childbirth—16.9%), the first-degree spontaneous trauma was found. In the case of VBAC, second-degree perineal spontaneous trauma was found (10.7%). A longer duration of the second stage of labor contributed to the occurrence of third- and fourth-degree perineal trauma (M = 40.1) and a shorter duration (M = 22.9) contributed to the occurrence of first-degree perineal lacerations. In addition, the second-degree perineal lacerations (M = 3583.3) occurred in the case of the larger birth weight of the neonate, and in the smaller birth weight of the neonate (M = 3526.5), first-degree trauma occurred. The above-mentioned relationships were statistically significant (*p* < 0.05). There were no significant relationships between the degrees of perineal laceration and the BMI index, the place of delivery, the use of oxytocin in the second stage of labor and the circumference of the neonatal head (*p* > 0.05)—Table 3.

Table 4 presents the analysis of logistic regression of factors predisposing to spontaneous perineal trauma during Childbirth The analysis of single-factor logistic regression showed eleven factors related to perineal laceration during childbirth, i.e., the age of the women giving birth, the BMI index before labor, the number of pregnancies and deliveries, the diagnosed gestational diabetes, home birth, the condition after cesarean section, the use of oxytocin in the second stage of labor, the duration of the second stage of labor, body weight, and the circumference of the neonatal head; however, the model of multivariable logistic regression analysis shows that the factors increasing the risk of perineal laceration during childbirth are the advanced age of women giving birth (β = 0.05; OR = 1.06; 95% CI 1.04–1.07; *p* < 0.05), VBAC (β = 0.40, OR = 1.49; 95% CI 1.14–1.93; *p* < 0.05), and higher birth weight of the neonate (β = 0.31; OR = 1.36; 95% CI 1.15–1.61; *p* < 0.05). On the other hand, the factors reducing the risk of spontaneous perineal trauma are a higher number of deliveries (β = −0.28; OR = 0.75; 95% CI 0.71–0.81; *p* < 0.05) and home birth (β = −0.20; OR = 0.82; 95% CI 0.72–0.91; *p* < 0.05).

Spontaneous trauma to the perineum during childbirth may have numerous consequences on the health of women and may affect their life and functioning [1,4,8,10], which is why it is a challenge for midwives and obstetricians during Childbirth Being aware of the factors influencing the occurrence of spontaneous perineal trauma, as well as experience and preparation for managing the childbirth, are important to minimize the risk of perineal trauma [6,12]. The above aspects were the basis for undertaking research aimed at analyzing the risk factors for spontaneous injuries of the birth canal soft tissues during non-operative delivery.

The result of the logistic regression analysis of our own research showed that the more advanced age of the woman giving birth predisposes her to spontaneous perineal trauma during childbirth (OR = 1.06). Research by Bodner-Adler et al. (2017) showed that, among others, maternal age was an independent risk factor for perineal trauma [27]. In addition, the analysis of the relationship between the degree of perineal laceration and the age of the giving birth showed that older respondents experienced a perineal laceration in the first degree, and younger ones experienced a perineal trauma in the third and fourth degree.

In turn, interesting research results in the field of risk factors for obstetric anal sphincter injury, which is a serious form of trauma/injury to the perineum following vaginal delivery, were presented by Nolan et al. (2021). They found that a maternal age over 35 has a protective effect on the perineum (OR = 0.68), which should be an important aspect of counseling pregnant women when deciding on the method of delivery [14], which may justify the results obtained by us. It should be emphasized here that the age of a pregnant/parturient woman is ever more often the subject of interest of researchers all over the world.

This is due to the more frequently observed phenomenon of pregnancies of women at an advanced age, i.e., 35 and over, which is often associated with numerous complications such as gestational diabetes mellitus, gestational hypertension, preeclampsia, preterm delivery, and cesarean section [28,29,30]. These observations may also explain our results—a single-factor logistic regression analysis showed that gestational diabetes in the woman giving birth increases the risk of spontaneous trauma to the perineum during childbirth.

Our own research shows that one of the factors influencing the occurrence of perineal laceration is the antenatal BMI of the mother. Reports on relationships between obesity and birth-related perineal trauma are contradictory. Some studies have reported a lower risk of obstetric anal sphincter injuries with increasing BMI in comparison with women with BMI within the normal range, while others have demonstrated a significant relationship between obesity and remarkable perineal trauma, whereby the probability of trauma is increased for obese women [15,16,17,18]. These discrepancies may result both from differences in the studied populations as well as from technical differences in childbirth management. In addition, the reasons for the less frequent occurrence of perineal injuries among obese women are not clearly justified. The literature on the subject shows that metabolic changes in these women may lead to a decrease in the contractility of the uterine, caused by reduced calcium flow or increased cholesterol levels in the uterine muscle tissue, and thus to a less rapid birth [31]. On the other hand, an association between high BMI during pregnancy and the occurrence of stretch marks in pregnant women, which in turn are associated with collagen abnormalities leading to flaccidity of the skin and its weaker mechanical properties, has been demonstrated [32].

Studies by MacArthur and MacArthur (2004) showed that perineal traumas were more often found in the primiparas, women after natural childbirth, and epidural anesthesia in the second stage of labor [33]. Similar results were obtained by Nolan et al. (2021), who showed that the strongest predictor of obstetric anal sphincter injury was nulliparity (OR = 4.20) [14]. In turn, the results of the research by Smith et al. (2013) showed that the adjusted OR for spontaneous trauma of the perineum was multiparity (OR = 0.42) [34]. An analysis of the logistic regression showed that a higher number of births is a protective factor in perineal laceration (OR = 0.75). In addition, the results of our own research on the analysis of the relationship between the degree of perineal laceration and the number of births showed that during the first birth, the perineal lacerations of the second and the next degree occurred more often, and during the second and the next birth, mainly spontaneous perineal injuries of the first stage occurred.

Analyses of our own research have shown that the place of childbirth is an important factor having a protective impact on the risk of spontaneous perineal laceration (OR = 0.82).

The results of the study by Bodner-Adler et al. (2017) showed that the lack of midwife care was also a significant risk factor for perineal trauma [27], which is of great importance. This is a significant variable that was not considered in our own study because all analyzed deliveries in our study were managed and delivered by midwives in accordance with the standard of care. In addition, this study can contribute to the provision of safe and evidence-based birth care, and at the same time, support the development of standards aimed at achieving positive outcomes of care in labor, including the effective protection of the perineum of the parturient woman [7]. In addition, the study included medical data from two areas where delivery could take place—the delivery room and the midwife-led unit. It should be emphasized that midwife-led units are not generally accessible and are not common places for giving birth in Poland. It should also be emphasized that the results of our own research showed that delivery in the midwifery-led unit was a factor in lowering the risk of spontaneous perineal injuries. Moreover, studies by Mizrachi et al. (2017) showed that with a greater experience of midwives, the risk of severe perineal injuries during childbirth decreases (OR = 0.95) [35]. The literature emphasizes that the supervision of low-risk births by midwives in freestanding midwife-led units can bring many benefits, primarily related to lower medicalization, limiting medical interventions such as those performed in the case of deliveries in hospital conditions [36], which confirms the results obtained by us.

Another important aspect analyzed in this paper was the VBAC and the perineal laceration. Studies by Elvander et al. (2019) showed that women undergoing vaginal birth after cesarean (VBAC) compared to nulliparous women have an increased risk of severe perineal injury (regardless of the indications and date of the primary cesarean section)—OR = 1.40 [19]. Uebergang et al. (2021) also emphasize that women after the first VBAC have a significantly increased risk of third- or fourth-degree perineal injuries compared to primiparous women that had natural labor [20]. The results of our own research are consistent with those presented above. Analysis of single-factor logistic regression showed that the VBAC increases the risk of spontaneous perineal injury (OR = 1.52). In addition, it was shown that in the case of VBAC, spontaneous second-degree perineal trauma was found most often. Elvander et al. (2019) presented the characteristics of women subjected to VBAC, who were much older, had lower height, and gave birth in a non-vertical position to neonates with a higher birth weight and with a larger head circumference [19] compared to nulliparous women, which may justify the above-mentioned research results. 

Oxytocin is one of the most used drugs in delivery rooms, used to induce or stimulate childbirth, which is the subject of interest to researchers around the world [37,38,39]. Nakai et al. (2006) conducted studies to learn about the incidence and risk factors of severe perineal trauma in Japanese patients. They found that the use of oxytocin to induce or stimulate childbirth is one of the important factors increasing the risk of severe perineal trauma [21]. The results of our own research are consistent because it has been shown, based on the analysis of single-factor logistic regression, that the use of oxytocin in the second stage of labor increases the risk of spontaneous perineal trauma. The important issue in the context of the use of oxytocin during natural childbirth is the caregiver because births with the participation of midwives are correlated with a smaller number of medical interventions, including perineal trauma, which is emphasized in the literature on the subject [36,40].

The second stage of childbirth is the most stressful part of the birth process (childbearing process). Decisions concerning the management of this stage, considering the optimal course for the mother and the child, have been under discussion for years [41]. According to the research, the longer duration of the second stage of childbirth is associated not only with an increased number of infections and the occurrence of postpartum hemorrhages but also with the occurrence of perineal trauma [22,42], which is also confirmed by the results of our own research. 

In addition to maternal and postpartum risk factors for perineal lacerations, the researchers also identified fetal–neonatal determinants. Komorowski et al. (2014) attempted to answer the question of whether a newborn’s large head increases the risk of perineal trauma or not. They found that in low-risk nulliparous women, a larger circumference of the baby’s head during labor increases the likelihood of perineal trauma; although the effect is small [8], similar results were obtained by Nóbrega et al. (2021) [23]. Bodner-Adler et al. (2017) showed that the independent risk factors for perineal trauma, apart from the mother’s age and the lack of midwife’s care, were also the diameter of the head and the birth weight of the newborn [27]. Moreover, the high birth weight of a child increases the risk of perineal laceration, which has been shown by numerous foreign studies: Dahlen et al. (2007)—OR = 2.3, D’Souza et al. (2020)—OR = 3.2, Smith et al. (2013)—OR = 1.001, Nóbrega et al. (2021)—OR = 3.42 [5,9,23,34], as well as confirmed by the results of our own research (OR = 1.36).

Identifying individual factors that may lead to spontaneous perineal trauma during non-operative delivery is difficult because, in the majority of women, several risk factors seem to interact. Experienced medical personnel taking care during childbirth should decide on the choice of perineal protection techniques based on both their own clinical judgment and the use of evidence-based medicine from current and reliable research and source materials: evidence-based medicine (EBM) as well as evidence-based midwifery practice (EBMP).

The strong part of our study is the large sample size, covering a long time period. In addition, several disruptive variables known to affect the risk of perineal injury were excluded from the analysis. We also did not include incomplete data in the analyses, which resulted in the exclusion of 1521 women from the study cohort. Another advantage is the quality of the collected data. Data have been collected from one institution, which reduces the risk of bias caused by differences in data collection or practices. Ultimately, the analyzed data included ethnically heterogeneous populations. 

However, the study we conducted has some limitations. It was a retrospective study that included only data contained in the hospital’s electronic medical records. This may result in non-uniformity regarding the documentation, keeping deficiencies in the documentation. The study was conducted based on the analysis of medical records from one center, which is also a limitation. In addition, despite a large number of respondents, on the basis of regression analysis, statistically significant predictors affecting perineal lacerations during delivery were found; however, the actual impact of the risk factor from OR is negligible. Data on preparation for childbirth by women, such as perineal massage, physical activity, or lifestyle, were not included.

It is necessary to conduct further research on the risk factors of spontaneous perineal injuries during childbirth, which will allow for updating the standards of childbirth management and, at the same time, improving the staff competencies of people responsible for the care and the quality-of-care improvement.

## 4. Conclusions

Birth age, BMI during childbirth, number of pregnancies and deliveries, diagnosed gestational diabetes, home birth, condition after cesarean section, use of oxytocin during the second stage of childbirth, duration of the second stage of childbirth, body weight, and circumference of the neonatal head are factors related to perineal laceration during childbirth.

Independent factors that increase the risk of perineal laceration during childbirth are the advanced age of the woman giving birth, the history of cesarean section, a higher birth weight of the newborn, and factors that reduce the risk of spontaneous perineal trauma are a higher number of deliveries and home birth.

The knowledge of the above factors can be used as part of preconception education when designing a birth plan with a specific patient and during delivery to avoid medically unjustified procedures that may generate a given complication. It is necessary to conduct further research on the issue of perineal trauma during childbirth to minimize their negative consequences.

## Figures and Tables

**Table 1:** Analysis of the dependencies between the occurrence of perineal laceration and maternal factors.

Variables	Study Group Laceration	Control Group No Laceration	*p*-Value
Age—M (SD)	31.9 (4.1)	31.2 (4.4)	0.000
Place of residence—*n* (%)			
City	6260 (86.5)	6760 (85.8)	0.220
Village	978 (13.5)	1119 (14.2)	
Education—*n* (%)			
Higher education	6482 (89.6)	6672 (84.7)	0.000
Secondary education	666 (9.2)	1003 (12.7)	
Primary education	90 (1.2)	204 (2.6)	
Marital status—*n* (%)			
In a relationship	6159 (85.1)	6346 (80.5)	0.000
Single	1079 (14.9)	1533 (19.5)	
BMI before labor—M (SD)	27.4 (7.0)	26.9 (5.6)	0.000
Gravidity—*n* (%)			
1	2162 (29.9)	2313 (29.4)	0.000
2	3060 (42.3)	3011 (38.2)	
3 and more	2016 (27.9)	2555 (32.4)	
Parity—*n* (%)			
1	2541 (35.1)	2667 (33.8)	0.000
2	3511 (48.5)	3424 (43.5)	
3 and more	1186 (16.4)	1788 (22.7)	
Gestational diabetes—*n* (%)			
No	6593 (91.1)	7248 (92.0)	0.046
Yes	645 (8.9)	631 (8.0)	
Pregnancy hypertension—*n* (%)			
No	7070 (97.7)	7729 (98.1)	0.074
Yes	168 (2.3)	150 (1.9)	
Pregnancy cholestasis—*n* (%)			
No	7208 (99.6)	7849 (99.6)	0.742
Yes	30 (0.4)	30 (0.4)	

M—mean; SD—standard deviation.

**Table 2:** Analysis of the dependencies between the occurrence of perineal laceration and perinatal and neonatal variables.

Variables	Study Group Laceration	Control Group No Laceration	*p*-Value
Place of labor—*n* (%)			
Hospital	5875 (81.2)	6128 (77.8)	0.000
Midwifery-led unit	1362 (18.8)	1751 (22.2)	
VBAC—*n* (%)			
Yes	358 (4.9)	299 (3.8)	0.001
No	6880 (95.1)	7580 (96.2)	
Oxytocin in 1st stage—*n* (%)			
Yes	46 (0.6)	43 (0.5)	0.471
No	7192 (99.4)	7836 (99.5)	
Oxytocin in 2nd stage—*n* (%)			
Yes	56 (0.8)	40 (0.5)	0.040
No	7182 (99.2)	7839 (99.5)	
Oxytocin in 1st and 2nd stages—*n* (%)			
Yes	753 (10.4)	766 (9.7)	0.164
No	6485 (89.6)	7113 (90.3)	
Epidural anesthesia—*n* (%)			
Yes	2154 (29.8)	2281 (29.0)	0.275
No	5084 (70.2)	5598 (71.0)	
Duration of 1st stage of labor (min.)—M (SD)	275.7 (148.7)	274.6 (147.0)	0.595
Duration of 2nd stage of labor (min.)—M (SD)	23.4 (17.9)	21.6 (18.2)	0.000
Apgar 1’—*n* (%)			
>7	7184 (99.3)	7796 (98.9)	0.046
≤7	54 (0.7)	83 (1.1)	
Apgar 5’—*n* (%)			
>7	7228 (99.9)	7863 (99.8)	0.336
≤7	10 (0.1)	16 (0.2)	
Apgar 10’—*n* (%)			
>7	7231 (99.9)	7869 (99.9)	0.580
≤7	7 (0.1)	10 (0.1)	
Birth Weight (g)—M (SD)	3529.1 (391.8)	3464.4 (397.3)	0.000
Head circumference (cm)—M (SD)	34.8 (1.7)	34.6 (1.7)	0.000

M—mean; SD—standard deviation; min—minutes; g—grams; cm—centimeters; VBAC—vaginal birth after cesarean.

**Table 3:** Analysis of the relationship between the degree of perineal laceration and selected variables.

Variables		Degree of Laceration		*p*-Value
	I Degree (N = 6910)	II Degree (N = 308)	III i IV Degree (N = 20)	
Age—M (SD)	31.9 (4.1)	30.9 (3.9)	29.9 (3.7)	0.000
BMI before labor—M (SD)	27.4 (7.2)	27.4 (3.9)	25.6 (3.4)	0.288
Gravidity—*n* (%)				
1	1964 (28.4)	184 (59.7)	14 (70.0)	0.000
2	2969 (43.0)	87 (28.2)	4 (20.0)	
3 and more	1977 (28.6)	37 (12.0)	2 (10.0)	
Parity—*n* (%)				
1	2323 (33.6)	203 (65.9)	15 (75.0)	0.000
2	3419 (49.5)	89 (28.9)	3 (15.0)	
3 and more	1168 (16.9)	16 (5.2)	2 (10.0)	
Place of labor—*n* (%)				
Hospital	5608 (81.2)	250 (81.2)	17 (85.0)	0.908
Midwifery-led unit	1302 (18.8)	58 (18.8)	3 (15.0)	
VBAC—*n* (%)				
Yes	324 (4.7)	33 (10.7)	1 (5.0)	0.000
No	6586 (95.3)	275 (89.3)	19 (95.0)	
Oxytocin in 2nd stage—*n* (%)				
Yes	50 (0.7)	6 (1.9)	0 (0.0)	0.052
No	6860 (99.3)	302 (98.1)	20 (100.0)	
Duration of 2nd stage of labor (min.)—M (SD)	22.9 (17.6)	33.3 (19.2)	40.1 (28.2)	0.000
Birth Weight (g)—M (SD)	3526.5 (391.5)	3583.3 (391.6)	3573.0 (439.9)	0.030
Head circumference (cm)—M (SD)	34.8 (1.7)	34.8 (1.2)	34.5 (1.7)	0.314

M—mean; SD—standard deviation; min—minutes; g—grams; cm—centimeters; VBAC—vaginal birth after cesarean.

**Table 4:** Logistic regression models of risk factors for perineal trauma during childbirth.

Variable	Univariate Logistic Regression				Multivariate Logistic Regression			
	β	OR	95% CI	*p*-Value	β	OR	95% CI	*p*-Value
Age (years)	0.04	1.04	1.03–1.05	0.000	0.05	1.06	1.04–1.07	0.000
BMI before labor	0.02	1.02	1.01–1.03	0.005	-	-	-	-
Number of pregnancies	−0.08	0.92	0.90–0.94	0.000	-	-	-	-
Number of deliveries	−0.15	0.86	0.83–0.89	0.000	−0.28	0.75	0.71–0.81	0.000
Gestational diabetes	0.12	1.12	1.00–1.26	0.046	-	-	-	-
Home of birth	−0.21	0.81	0.75–0.88	0.000	−0.20	0.82	0.72–0.91	0.000
Vaginal birth after cesarean	0.28	1.32	1.13–1.54	0.001	0.40	1.49	1.14–1.93	0.002
Oxytocin in 2nd stage	0.42	1.53	1.02–2.30	0.041	-	-	-	-
Duration of 2nd stage of labor	0.01	1.01	1.00–1.01	0.000	-	-	-	-
Birth Weight	0.31	1.37	1.23–1.52	0.000	0.31	1.36	1.15–1.61	0.000
Head circumference	0.06	1.06	1.04–1.08	0.000	-	-	-	-

## Data Availability

The data presented in this study are available on request from the corresponding author.

## References

[1] Huang J., Lu H., Zang Y., Ren L., Li C., Wang J. (2020). The effects of hands on and hands off/poised techniques on maternal outcomes: A systematic review and meta-analysis. Midwifery.

[2] Webb S., Sherburn M., Ismail K.M.K. (2014). Managing perineal trauma after Childbirth. BMJ.

[3] O’Kelly S.M., Moore Z.E. (2017). Antenatal maternal education for improving postnatal perineal healing for women who have birthed in a hospital setting. Cochrane Database Syst. Rev..

[4] Jones K., Webb S., Manresa M., Hodgetts-Morton V., Morris R.K. (2019). The incidence of wound infection and dehiscence following childbirth-related perineal trauma: A systematic review of the evidence. Eur. J. Obstet. Gynecol. Reprod. Biol..

[5] Dahlen H.G., Ryan M., Homer C.S.E., Cooke M. (2007). An Australian prospective cohort study of risk factors for severe perineal trauma during childbirth. Midwifery.

[6] East C.E., Lau R., Biro M.A. (2015). Midwives’ and doctors’ perceptions of their preparation for and practice in managing the perineum in the second stage of labour: A cross-sectional survey. Midwifery.

[7] De Souza M.R.T., Farias L.M.V.C., Ribeiro G.L., Coelho T.D.S., da Costa C.C., Damasceno A.K.C. (2020). Factors related to perineal outcome after vaginal delivery in primiparas: A cross-sectional study. Rev. Esc. Enferm. USP.

[8] Komorowski L.K., Leeman L.M., Fullilove A.M., Bedrick E.J., Migliaccio L.D., Rogers R.G. (2014). Does a large infant head or a short perineal body increase the risk of obstetrical perineal trauma?. Birth.

[9] D’Souza J.C., Monga A., Tincello D.G. (2020). Risk factors for perineal trauma in the primiparous population during non-operative vaginal delivery. Int. Urogynecol. J..

[10] Smith V., Guilliland K., Dixon L., Reilly M., Keegan C., McCann C., Begley C. (2017). Irish and New Zealand Midwives’ expertise at preserving the perineum intact (the MEPPI study): Perspectives on preparations for birth. Midwifery.

[11] Hsieh C.-H., Chen C.-L., Han T.-J., Lin P.-J., Chiu H.-C. (2018). Factors Influencing Postpartum Fatigue in Vaginal-Birth Women: Testing a Path Model. J. Nurs. Res..

[12] Cronin R.S., Li M., Culliney K., Maude R., Nelson K. (2018). Midwifery management of second-degree perineal tears in New Zealand: A cross-sectional survey of practice. Women Birth..

[13] Tsakiridis I., Mamopoulos A., Athanasiadis A., Dagklis T. (2018). Obstetric Anal Sphincter Injuries at Vaginal Delivery: A Review of Recently Published National Guidelines. Obstet. Gynecol. Surv..

[14] Nolan C.E., O’leary B.D., Ciprike V. (2021). Is the older perineum a safer perineum? Risk factors for obstetric anal sphincter injury. Ir. J. Med. Sci..

[15] Lindholm E.S., Altman D. (2013). Risk of obstetric anal sphincter lacerations among obese women. BJOG.

[16] Blomberg M. (2014). Maternal body mass index and risk of obstetric anal sphincter injury. BioMed Res. Int..

[17] Meister M.R., Cahill A.G., Conner S.N., Woolfolk C.L., Lowder J.L. (2016). Predicting obstetric anal sphincter injuries in a modern obstetric population. Am. J. Obstet. Gynecol..

[18] Drusany S.D., Bukovec P., Jakopič K., Zdravevski E., Trajkovik V., Lukanović A. (2017). Can we predict obstetric anal sphincter injury?. Eur. J. Obstet. Gynecol. Reprod. Biol..

[19] Elvander C., Ahlberg M., Edqvist M., Stephansson O. (2019). Severe perineal trauma among women un-dergoing vaginal birth after cesarean delivery: A population-based cohort study. Birth.

[20] Uebergang J., Hiscock R., Hastie R., Middleton A., Pritchard N., Walker S., Tong S., Lindquist A. (2022). Risk of obstetric anal sphincter injury among women who birth vaginally after a prior caesarean section: A state-wide cohort study. BJOG.

[21] Nakai A., Yoshida A., Yamaguchi S., Kawabata I., Hayashi M., Yokota A., Isozaki T., Takeshita T. (2006). Incidence and risk factors for severe perineal laceration after vaginal delivery in Japanese patients. Arch. Gynecol. Obstet..

[22] Simic M., Cnattingius S., Petersson G., Sandström A., Stephansson O. (2017). Duration of second stage of labor and instrumental delivery as risk factors for severe perineal lacerations: Population-based study. BMC Pregnancy Childbirth.

[23] Nóbrega M.A., Pereira G.M.V., Brito L.G.O., Luz A.G., Lajos G.J. (2021). Severe Perineal Trauma in a Brazilian Southeastern Tertiary Hospital: A Retrospective Cohort Study. Female Pelvic Med. Reconstr. Surg..

[24] Hauck Y.L., Lewis L., Nathan E.A., White C., Doherty D.A. (2015). Risk factors for severe perineal trauma during vaginal childbirth: A Western Australian retrospective cohort study. Women Birth..

[25] Klokk R., Bakken K.S., Markestad T., Holten-Andersen M.N. (2022). Modifiable and non-modifiable risk factors for obstetric anal sphincter injury in a Norwegian Region: A case-control study. BMC Pregnancy Childbirth.

[26] Von Elm E., Altman D.G., Egger M., Pocock S.J., Gøtzsche P.C., Vandenbroucke J.P., Strobe I. (2007). The Strengthening the Reporting of Observational Studies in Epidemiology (STROBE) Statement: Guidelines for Reporting Observational Studies. Bull. World Health Organ..

[27] Bodner-Adler B., Kimberger O., Griebaum J., Husslein P., Bodner K. (2017). A ten-year study of midwife-led care at an Austrian tertiary care center: A retrospective analysis with special consideration of perineal trauma. BMC Pregnancy Childbirth.

[28] Lean S.C., Derricott H., Jones R.L., Heazell A.E.P. (2017). Advanced maternal age and adverse pregnancy outcomes: A systematic review and meta-analysis. PLoS ONE.

[29] Kahveci B., Melekoglu R., Evruke I.C., Cetin C. (2018). The effect of advanced maternal age on perinatal outcomes in nulliparous singleton pregnancies. BMC Pregnancy Childbirth.

[30] Pinheiro R.L., Areia A.L., Pinto A.M., Donato H. (2019). Advanced maternal age: Adverse outcomes of pregnancy, a meta-analysis. Acta Med. Port..

[31] Zhang J., Kendrick A., Quenby S., Wray S. (2007). Contractility and calcium signaling of human myometrium are profoundly affected by cholesterol manipulation: Implications for labor?. Reprod. Sci..

[32] Halperin O., Raz I., Ben-Gal L., Or-Chen K., Granot M. (2010). Prediction of perineal trauma during childbirth by assessment of striae gravidarum score. J. Obstet. Gynecol. Neonatal Nurs..

[33] MacArthur A.J., MacArthur C. (2004). Incidence, severity, and determinants of perineal pain after vaginal delivery: A prospective cohort study. Am. J. Obstet. Gynecol..

[34] Smith L.A., Price N., Simonite V., Burns E.E. (2013). Incidence of and risk factors for perineal trauma: A prospective observational study. BMC Pregnancy Childbirth.

[35] Mizrachi Y., Leytes S., Levy M., Hiaev Z., Ginath S., Bar J., Kovo M. (2017). Does midwife experience affect the rate of severe perineal tears?. Birth.

[36] Bączek G., Tataj-Puzyna U., Sys D., Baranowska B. (2020). Freestanding midwife-led units: A narrative review. Iran. J. Nurs. Midwifery Res..

[37] Selin L., Berg M., Wennerholm U.B., Dencker A. (2021). Dosage of oxytocin for augmentation of labor and women’s childbirth experiences: A randomized controlled trial. Acta Obstet. Gynecol. Scand..

[38] Nicola L., Yang J., Egger M.J., Nygaard I.E. (2021). Effects of Oxytocin for Induction and Augmentation of Labor on Pelvic Floor Symptoms and Support in the Postpartum Period. Female Pelvic Med. Reconstr. Surg..

[39] Baranowska B., Kajdy A., Kiersnowska I., Sys D., Tataj-Puzyna U., Daly D., Rabijewski M., Bączek G., Węgrzynowska M. (2021). Oxytocin administration for induction and augmentation of labour in polish maternity units—An observational study. BMC Pregnancy Childbirth.

[40] Browne M., Jacobs M., Lahiff M., Miller S. (2010). Perineal injury in nulliparous women giving birth at a community hospital: Reduced risk in births attended by certified nurse-midwives. J. Midwifery Womens Health.

[41] Kopas M.L. (2014). A review of evidence-based practices for management of the second stage of labor. J. Midwifery Womens Health.

[42] Rouse D.J., Weiner S.J., Bloom S.L., Varner M.W., Spong C.Y., Ramin S.M., Caritis S.N., Peaceman A.M., Sorokin Y., Malone F.D. (2009). Second-stage labor duration in nulliparous women: Relationship to maternal and perinatal outcomes. Am. J. Obstet. Gynecol..

